# Crystal structure of Ti_4_Ni_2_C

**DOI:** 10.1107/S2414314624000439

**Published:** 2024-01-19

**Authors:** Huizi Liu, Xinyu Liang, Yibo Liu, Changzeng Fan, Bin Wen, Lifeng Zhang

**Affiliations:** a State Key Laboratory of Metastable Materials Science and Technology, Yanshan University, Qinhuangdao 066004, People’s Republic of China; b Hebei Key Lab for Optimizing Metal Product Technology and Performance, Yanshan University, Qinhuangdao 066004, People’s Republic of China; c School of Mechanical and Materials Engineering, North China University of Technology, Beijing 100144, People’s Republic of China; Vienna University of Technology, Austria

**Keywords:** crystal structure, high-pressure sinter­ing, inter­metallic, Ti_4_Ni_2_C phase

## Abstract

Single crystals of the inter­metallic phase with composition Ti_4_Ni_2_C were serendipitously obtained by high-pressure sinter­ing of a mixture with initial chemical composition Ti_2_Ni. The Ti_4_Ni_2_C phase crystallizes in the *Fd*  *m* space group and can be considered as a partially filled Ti_2_Ni structure with the C atom occupying an octa­hedral void. Ti_4_Ni_2_C is isotypic with Ti_4_Ni_2_O, Nb_4_Ni_2_C and Ta_4_Ni_2_C, all of which were studied previously by means of powder diffraction.

## Abstract 2

Ti_4_Ni_2_C crystallizes isotypically with Ti_4_Ni_2_O, Nb_4_Ni_2_C and Ta_4_Ni_2_C and can be considered as a partially filled Ti_2_Ni structure with the C atom occupying an octa­hedral void.

## Graphical abstract

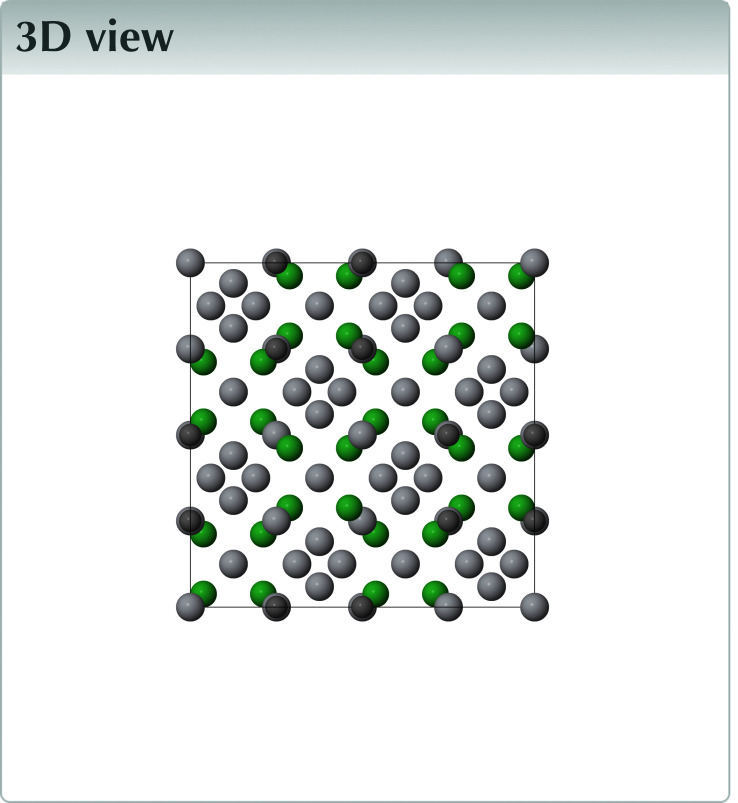

## Structure description

A large number of inter­metallic phases can be grouped into classes of compounds based on structural or chemical similarities. For example, Mueller & Knott (1963 ▸) investigated the related crystal structures of Ti_2_Cu, Ti_2_Ni, Ti_4_Ni_2_O and Ti_4_Cu_2_O by X-ray and neutron powder diffraction. They determined that the Ti_2_Ni phase crystallizes in the *Fd* 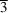 *m* space group, with cell parameter *a* = 11.3193 (2) Å and with 96 atoms per unit cell; the Ti_4_Ni_2_O (Ti_4_Cu_2_O) phase also crystallizes in the *Fd* 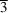 *m* space group, with cell parameter *a* = 11.3279 (1) Å [*a* = 11.4353 (2) Å] and with 112 atoms per unit cell. The latter phases can be considered as partially filled Ti_2_Ni variants with the additional oxygen atom occupying an octa­hedral position. Holleck & Thummler (1967 ▸) studied a series of carbides, nitrides and oxides in ternary systems and reported that Nb_4_Ni_2_C (*a* = 11.64 Å) and Ta_4_Ni_2_C (*a* = 11.61 Å) crystallize in the same partially filled Ti_2_Ni structure. Sadrnezhaad *et al.* (2009 ▸) and Shigeo *et al.* (1993 ▸) have confirmed the existence of the Ti_4_Ni_2_C phase. However, no detailed study has been performed so far with respect to the determination of its crystal structure.

In the present study, the crystal structure model of Ti_4_Ni_2_C has been refined on the basis of single-crystal X-ray diffraction data. The lattice parameter *a* is similar to those of previously reported isotypic phases (see above), and its chemical composition was refined to be exactly Ti_4_Ni_2_C in accordance with the EDX results (see Fig. S1 and Table S1 in the supporting information). Carbon present in the crystal structure most likely originated from the graphite crucible used during high pressure sinter­ing (HPS).

**Figure 1:**
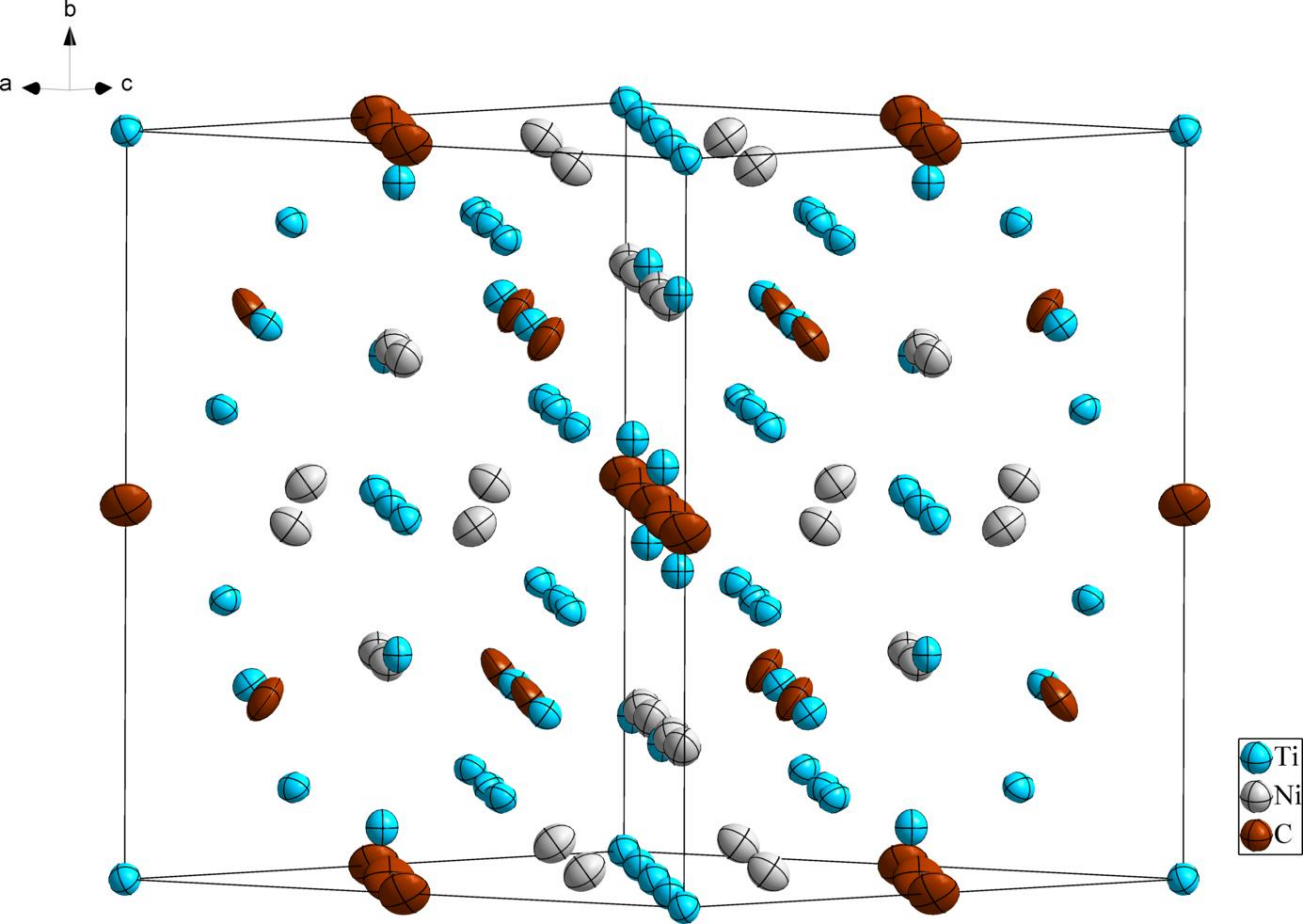
The crystal structure of Ti_4_Ni_2_C (one unit cell), with displacement ellipsoids drawn at the 99% probability level.

**Figure 2:**
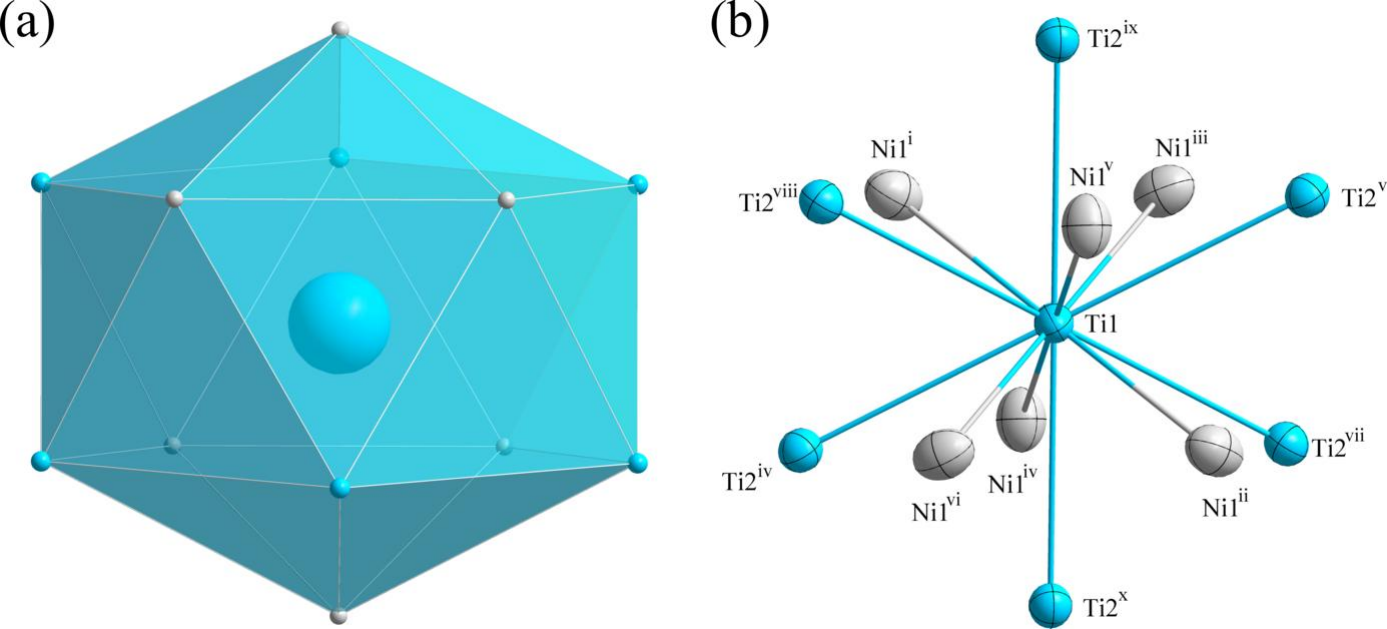
(*a*) The icosa­hedron formed around the Ti1 atom at the 16 *c* site; (*b*) the environment of the Ti1 atom with displacement ellipsoids given at the 99% probability level. [Symmetry codes: (i) *x* − 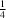 , −*y*, *z* − 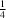 ; (ii) −*x* + 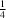 , *y*, −*z* + 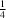 ; (iii) −*x*, *y* − 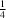 , *z* − 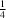 ; (iv) *x* − 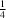 , *y* − 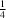 , −*z*; (v) −*x* + 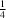 , −*y* + 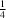 , *z*; (vi) *x*, −*y* + 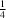 , −*z* + 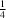 ; (vii) −*z*, *x* − 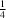 , *y* − 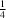 ; (viii) *z*, −*x* + 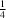 , −*y* + 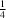 ; (ix) *y* − 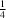 , −*z*, *x* − 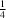 ; (*x*) −*y* + 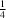 , *z*, −*x* + 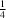 .]

Ti_4_Ni_2_C crystallizes isotypically with other Ti_4_Ni_2_ *X* compounds (*X* = C, N, O) with a partially filled Ti_2_Ni structure in space group type *Fd* 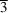 *m*. Fig. 1 ▸ shows the distribution of the atoms in the unit cell of Ti_4_Ni_2_C. The environments of the Ti1 and C1 sites are shown in Figs. 2 ▸ and 3 ▸, respectively. The Ti1 atom is situated at a position with site symmetry . 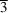 *m* (multiplicity 16, Wyckoff letter *c*). It is surrounded by six Ti2 atoms (2*.mm*, 48*f*) and six Ni1 atoms (.3*m*; 32*e*), defining the center of an icosa­hedron. The C1 atom occupies a position with site symmetry . 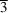 *m* (16*d*) and centers an octa­hedron defined by six Ti2 atoms. The shortest Ti1⋯Ti2 separation is 2.9415 (9) Å and the shortest Ti1⋯Ni1 separation is 2.4750 (4) Å; the C1—Ti2 bond length is 2.1127 (4) Å.

## Synthesis and crystallization

The high-purity elements Ti (indicated purity 99.5%; 0.6291 g) and Ni (indicated purity 99.9%; 0.3869 g) were mixed uniformly in the stoichiometric ratio 2:1 and thoroughly ground in an agate mortar. The blended powders were then placed in a cemented carbide grinding mould of 5 mm diameter, and pressed into a tablet at about 4 MPa for 1 min. A cylindrical block was obtained without deformations or cracks. Details of the high-pressure sinter­ing experiment using a six-anvil high-temperature high-pressure apparatus can be found elsewhere (Liu & Fan, 2018 ▸). The samples were pressurized up to 6 GPa and heated to 1573 K for 40 min, and then rapidly cooled to room temperature by turning off the furnace power. A piece of a single-crystal (0.06×0.06×0.04 mm^3^) was selected and mounted on a glass fibre for SXRD measurements.

## Refinement

Crystal data, data collection and structure refinement details are summarized in Table 1 ▸. For better comparison, the labeling scheme and atomic coordinates of Ti_4_Ni_2_C were adapted from Nb_4_Ni_2_C and Ta_4_Ni_2_C (Holleck & Thuemmler, 1967 ▸). The maximum and minimum residual electron densities in the final difference map are located 1.10 Å from site Ni1 and 0.17 Å from Ti2, respectively.

## Supplementary Material

Crystal structure: contains datablock(s) I. DOI: 10.1107/S2414314624000439/wm4205sup1.cif

Structure factors: contains datablock(s) I. DOI: 10.1107/S2414314624000439/wm4205Isup2.hkl

Supporting information file. DOI: 10.1107/S2414314624000439/wm4205sup3.docx

CCDC reference: 2324933

Additional supporting information:  crystallographic information; 3D view; checkCIF report

## Figures and Tables

**Figure 3:**
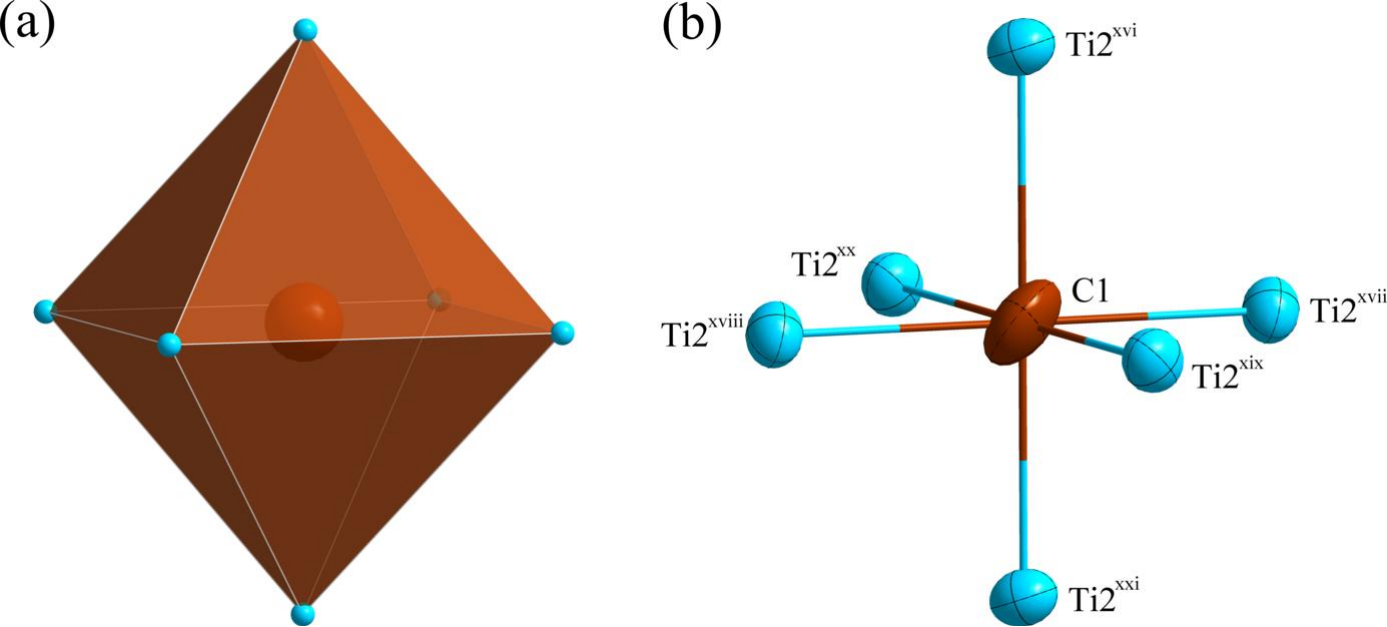
(*a*) The octa­hedron formed around the C1 atom at the 16 *d* site; (*b*) the environment of the C1 atom with displacement ellipsoids given at the 99% probability level. [Symmetry codes:·(xvi) −*y* + 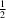 , −*z* + 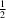 , −*x* + 1; (xvii) *x*, *y* + 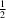 , *z* + 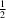 ; (xviii) −*x* + 1, −*y* + 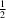 , −*z* + 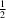 ; (xix) *z* + 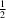 , *x*, *y* + 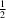 ; (xx) −*z* + 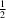 , −*x* + 1, −*y* + 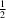 ; (xxi) *y* + 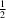 , *z* + 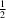 , *x*.]

**Table 1:** Experimental details

Crystal data	
Chemical formula	Ti_4_Ni_2_C
*M*_r_	320.87
Crystal system, space group	Cubic, *F* *d* 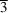 *m*
Temperature (K)	296
*a* (Å)	11.3235 (8)
*V* (Å^3^)	1451.9 (3)
*Z*	16
Radiation type	Mo *K*α
μ (mm^−1^)	18.28
Crystal size (mm)	0.06 × 0.06 × 0.04

Data collection	
Diffractometer	Bruker D8 Venture Photon 100 CMOS
Absorption correction	Multi-scan (*SADABS*; Krause *et al.*, 2015 ▸)
*T*_min_, *T*_max_	0.520, 0.746
No. of measured, independent and observed [*I* > 2σ(*I*)] reflections	12231, 105, 97
*R*_int_	0.091
(sin θ/λ)_max_ (Å^−1^)	0.649

Refinement	
*R*[*F* ^2^ > 2σ(*F* ^2^)], *wR*(*F* ^2^), *S*	0.024, 0.051, 1.22
No. of reflections	105
No. of parameters	12
Δρ_max_, Δρ_min_ (e Å^−3^)	0.48, −0.70

Computer programs: *APEX3* (Bruker, 2015 ▸), *SAINT* (Bruker, 2015 ▸), *SHELXT* (Sheldrick, 2015*a* ▸), *SHELXL* (Sheldrick, 2015*b* ▸), *DIAMOND* (Brandenburg & Putz, 2017 ▸) and *publCIF* (Westrip, 2010 ▸).

## full crystallographic data

### Crystal data

**Table:**

Ti_4_Ni_2_C	Mo *K*α radiation, λ = 0.71073 Å
*M**_r_* = 320.87	Cell parameters from 3145 reflections
Cubic, *F**d*3*m*	θ = 3.1–27.5°
*a* = 11.3235 (8) Å	µ = 18.28 mm^−^^1^
*V* = 1451.9 (3) Å^3^	*T* = 296 K
*Z* = 16	Lump, gray
*F*(000) = 2400	0.06 × 0.06 × 0.04 mm
*D*_x_ = 5.875 Mg m^−^^3^	

### Data collection

**Table:**

Bruker D8 Venture Photon 100 CMOS diffractometer	97 reflections with *I* > 2σ(*I*)
phi and ω scans	*R*_int_ = 0.091
Absorption correction: multi-scan (SADABS; Krause *et al.*, 2015)	θ_max_ = 27.5°, θ_min_ = 3.1°
*T*_min_ = 0.520, *T*_max_ = 0.746	*h* = −14→14
12231 measured reflections	*k* = −14→14
105 independent reflections	*l* = −14→14

### Refinement

**Table:**

Refinement on *F*^2^	12 parameters
Least-squares matrix: full	0 restraints
*R*[*F*^2^ > 2σ(*F*^2^)] = 0.024	*w* = 1/[σ^2^(*F*_o_^2^) + (0.0226*P*)^2^ + 31.699*P*] where *P* = (*F*_o_^2^ + 2*F*_c_^2^)/3
*wR*(*F*^2^) = 0.051	(Δ/σ)_max_ < 0.001
*S* = 1.22	Δρ_max_ = 0.48 e Å^−^^3^
105 reflections	Δρ_min_ = −0.70 e Å^−^^3^

### Special details

**Table:**

Geometry. All esds (except the esd in the dihedral angle between two l.s. planes) are estimated using the full covariance matrix. The cell esds are taken into account individually in the estimation of esds in distances, angles and torsion angles; correlations between esds in cell parameters are only used when they are defined by crystal symmetry. An approximate (isotropic) treatment of cell esds is used for estimating esds involving l.s. planes.

### Fractional atomic coordinates and isotropic or equivalent isotropic displacement parameters (Å^2^)

**Table:**

	*x*	*y*	*z*	*U*_iso_*/*U*_eq_	
Ti1	0.000000	0.000000	0.000000	0.0054 (5)	
Ni1	0.21179 (6)	0.21179 (6)	0.21179 (6)	0.0080 (3)	
Ti2	0.44034 (11)	0.125000	0.125000	0.0052 (3)	
C1	0.500000	0.500000	0.500000	0.010 (3)	

### Atomic displacement parameters (Å^2^)

**Table:**

	*U*^11^	*U*^22^	*U*^33^	*U*^12^	*U*^13^	*U*^23^
Ti1	0.0054 (5)	0.0054 (5)	0.0054 (5)	0.0004 (5)	0.0004 (5)	0.0004 (5)
Ni1	0.0080 (3)	0.0080 (3)	0.0080 (3)	0.0011 (2)	0.0011 (2)	0.0011 (2)
Ti2	0.0063 (6)	0.0046 (4)	0.0046 (4)	0.000	0.000	−0.0005 (4)
C1	0.010 (3)	0.010 (3)	0.010 (3)	−0.003 (3)	−0.003 (3)	−0.003 (3)

### Geometric parameters (Å, º)

**Table:**

Ti1—Ni1^i^	2.4750 (4)	Ni1—Ti2^xiii^	2.6249 (9)
Ti1—Ni1^ii^	2.4750 (4)	Ni1—Ni1^v^	2.7797 (18)
Ti1—Ni1^iii^	2.4750 (4)	Ni1—Ni1^vi^	2.7797 (18)
Ti1—Ni1^iv^	2.4750 (4)	Ni1—Ni1^ii^	2.7797 (18)
Ti1—Ni1^v^	2.4750 (4)	Ni1—Ti2^xiv^	2.9376 (11)
Ti1—Ni1^vi^	2.4750 (4)	Ni1—Ti2^xv^	2.9376 (11)
Ti1—Ti2^vii^	2.9415 (9)	Ni1—Ti2	2.9376 (11)
Ti1—Ti2^viii^	2.9415 (9)	Ti2—C1^xvi^	2.1127 (4)
Ti1—Ti2^ix^	2.9415 (9)	Ti2—C1^xvii^	2.1127 (4)
Ti1—Ti2^iv^	2.9415 (9)	Ti2—Ti2^xviii^	2.9571 (17)
Ti1—Ti2^v^	2.9415 (9)	Ti2—Ti2^xix^	2.9571 (17)
Ti1—Ti2^x^	2.9415 (9)	Ti2—Ti2^xx^	2.9571 (17)
Ni1—Ti2^xi^	2.6249 (9)	Ti2—Ti2^xxi^	2.9571 (17)
Ni1—Ti2^xii^	2.6249 (9)		
			
Ni1^i^—Ti1—Ni1^ii^	180.00 (4)	Ti2^xiii^—Ni1—Ti2^xiv^	65.437 (12)
Ni1^i^—Ti1—Ni1^iii^	68.33 (4)	Ni1^v^—Ni1—Ti2^xiv^	61.76 (2)
Ni1^ii^—Ti1—Ni1^iii^	111.67 (4)	Ni1^vi^—Ni1—Ti2^xiv^	112.73 (2)
Ni1^i^—Ti1—Ni1^iv^	68.33 (4)	Ni1^ii^—Ni1—Ti2^xiv^	112.73 (2)
Ni1^ii^—Ti1—Ni1^iv^	111.67 (4)	Ti1^vi^—Ni1—Ti2^xv^	65.185 (5)
Ni1^iii^—Ti1—Ni1^iv^	68.33 (4)	Ti1^v^—Ni1—Ti2^xv^	65.185 (5)
Ni1^i^—Ti1—Ni1^v^	111.67 (4)	Ti1^ii^—Ni1—Ti2^xv^	166.08 (5)
Ni1^ii^—Ti1—Ni1^v^	68.33 (4)	Ti2^xi^—Ni1—Ti2^xv^	65.437 (12)
Ni1^iii^—Ti1—Ni1^v^	111.67 (4)	Ti2^xii^—Ni1—Ti2^xv^	123.55 (5)
Ni1^iv^—Ti1—Ni1^v^	180.00 (6)	Ti2^xiii^—Ni1—Ti2^xv^	65.437 (12)
Ni1^i^—Ti1—Ni1^vi^	111.67 (4)	Ni1^v^—Ni1—Ti2^xv^	112.73 (2)
Ni1^ii^—Ti1—Ni1^vi^	68.33 (4)	Ni1^vi^—Ni1—Ti2^xv^	112.73 (2)
Ni1^iii^—Ti1—Ni1^vi^	180.0	Ni1^ii^—Ni1—Ti2^xv^	61.76 (2)
Ni1^iv^—Ti1—Ni1^vi^	111.67 (4)	Ti2^xiv^—Ni1—Ti2^xv^	118.526 (10)
Ni1^v^—Ti1—Ni1^vi^	68.33 (4)	Ti1^vi^—Ni1—Ti2	166.08 (5)
Ni1^i^—Ti1—Ti2^vii^	122.80 (3)	Ti1^v^—Ni1—Ti2	65.185 (5)
Ni1^ii^—Ti1—Ti2^vii^	57.20 (3)	Ti1^ii^—Ni1—Ti2	65.185 (5)
Ni1^iii^—Ti1—Ti2^vii^	65.020 (15)	Ti2^xi^—Ni1—Ti2	65.437 (12)
Ni1^iv^—Ti1—Ti2^vii^	65.020 (15)	Ti2^xii^—Ni1—Ti2	65.437 (12)
Ni1^v^—Ti1—Ti2^vii^	114.980 (15)	Ti2^xiii^—Ni1—Ti2	123.55 (5)
Ni1^vi^—Ti1—Ti2^vii^	114.980 (15)	Ni1^v^—Ni1—Ti2	112.73 (2)
Ni1^i^—Ti1—Ti2^viii^	57.20 (3)	Ni1^vi^—Ni1—Ti2	61.76 (2)
Ni1^ii^—Ti1—Ti2^viii^	122.80 (3)	Ni1^ii^—Ni1—Ti2	112.73 (2)
Ni1^iii^—Ti1—Ti2^viii^	114.980 (15)	Ti2^xiv^—Ni1—Ti2	118.525 (10)
Ni1^iv^—Ti1—Ti2^viii^	114.980 (15)	Ti2^xv^—Ni1—Ti2	118.525 (10)
Ni1^v^—Ti1—Ti2^viii^	65.020 (15)	C1^xvi^—Ti2—C1^xvii^	142.70 (6)
Ni1^vi^—Ti1—Ti2^viii^	65.020 (15)	C1^xvi^—Ti2—Ni1^xxii^	88.304 (12)
Ti2^vii^—Ti1—Ti2^viii^	180.00 (3)	C1^xvii^—Ti2—Ni1^xxii^	88.304 (12)
Ni1^i^—Ti1—Ti2^ix^	65.020 (15)	C1^xvi^—Ti2—Ni1^xxiii^	88.304 (12)
Ni1^ii^—Ti1—Ti2^ix^	114.980 (15)	C1^xvii^—Ti2—Ni1^xxiii^	88.304 (12)
Ni1^iii^—Ti1—Ti2^ix^	65.020 (15)	Ni1^xxii^—Ti2—Ni1^xxiii^	169.38 (6)
Ni1^iv^—Ti1—Ti2^ix^	122.80 (3)	C1^xvi^—Ti2—Ni1^vi^	136.89 (5)
Ni1^v^—Ti1—Ti2^ix^	57.20 (3)	C1^xvii^—Ti2—Ni1^vi^	80.41 (3)
Ni1^vi^—Ti1—Ti2^ix^	114.980 (15)	Ni1^xxii^—Ti2—Ni1^vi^	94.68 (3)
Ti2^vii^—Ti1—Ti2^ix^	118.270 (7)	Ni1^xxiii^—Ti2—Ni1^vi^	94.68 (3)
Ti2^viii^—Ti1—Ti2^ix^	61.730 (7)	C1^xvi^—Ti2—Ni1	80.41 (3)
Ni1^i^—Ti1—Ti2^iv^	65.020 (15)	C1^xvii^—Ti2—Ni1	136.89 (5)
Ni1^ii^—Ti1—Ti2^iv^	114.980 (15)	Ni1^xxii^—Ti2—Ni1	94.68 (3)
Ni1^iii^—Ti1—Ti2^iv^	122.80 (3)	Ni1^xxiii^—Ti2—Ni1	94.68 (3)
Ni1^iv^—Ti1—Ti2^iv^	65.020 (15)	Ni1^vi^—Ti2—Ni1	56.48 (5)
Ni1^v^—Ti1—Ti2^iv^	114.980 (15)	C1^xvi^—Ti2—Ti1^ii^	103.550 (18)
Ni1^vi^—Ti1—Ti2^iv^	57.20 (3)	C1^xvii^—Ti2—Ti1^ii^	103.550 (18)
Ti2^vii^—Ti1—Ti2^iv^	118.270 (7)	Ni1^xxii^—Ti2—Ti1^ii^	138.19 (4)
Ti2^viii^—Ti1—Ti2^iv^	61.730 (7)	Ni1^xxiii^—Ti2—Ti1^ii^	52.426 (19)
Ti2^ix^—Ti1—Ti2^iv^	118.270 (7)	Ni1^vi^—Ti2—Ti1^ii^	49.79 (2)
Ni1^i^—Ti1—Ti2^v^	114.980 (15)	Ni1—Ti2—Ti1^ii^	49.79 (2)
Ni1^ii^—Ti1—Ti2^v^	65.020 (15)	C1^xvi^—Ti2—Ti1^v^	103.550 (18)
Ni1^iii^—Ti1—Ti2^v^	57.20 (3)	C1^xvii^—Ti2—Ti1^v^	103.550 (18)
Ni1^iv^—Ti1—Ti2^v^	114.980 (15)	Ni1^xxii^—Ti2—Ti1^v^	52.426 (19)
Ni1^v^—Ti1—Ti2^v^	65.020 (15)	Ni1^xxiii^—Ti2—Ti1^v^	138.19 (4)
Ni1^vi^—Ti1—Ti2^v^	122.80 (3)	Ni1^vi^—Ti2—Ti1^v^	49.79 (2)
Ti2^vii^—Ti1—Ti2^v^	61.730 (7)	Ni1—Ti2—Ti1^v^	49.79 (2)
Ti2^viii^—Ti1—Ti2^v^	118.270 (7)	Ti1^ii^—Ti2—Ti1^v^	85.77 (3)
Ti2^ix^—Ti1—Ti2^v^	61.730 (7)	C1^xvi^—Ti2—Ti2^xviii^	45.58 (2)
Ti2^iv^—Ti1—Ti2^v^	180.00 (3)	C1^xvii^—Ti2—Ti2^xviii^	104.34 (3)
Ni1^i^—Ti1—Ti2^x^	114.980 (15)	Ni1^xxii^—Ti2—Ti2^xviii^	115.62 (3)
Ni1^ii^—Ti1—Ti2^x^	65.020 (15)	Ni1^xxiii^—Ti2—Ti2^xviii^	55.72 (2)
Ni1^iii^—Ti1—Ti2^x^	114.980 (15)	Ni1^vi^—Ti2—Ti2^xviii^	149.263 (5)
Ni1^iv^—Ti1—Ti2^x^	57.20 (3)	Ni1—Ti2—Ti2^xviii^	112.73 (2)
Ni1^v^—Ti1—Ti2^x^	122.80 (3)	Ti1^ii^—Ti2—Ti2^xviii^	100.245 (14)
Ni1^vi^—Ti1—Ti2^x^	65.020 (15)	Ti1^v^—Ti2—Ti2^xviii^	149.135 (4)
Ti2^vii^—Ti1—Ti2^x^	61.730 (7)	C1^xvi^—Ti2—Ti2^xix^	45.58 (2)
Ti2^viii^—Ti1—Ti2^x^	118.270 (7)	C1^xvii^—Ti2—Ti2^xix^	104.34 (3)
Ti2^ix^—Ti1—Ti2^x^	180.0	Ni1^xxii^—Ti2—Ti2^xix^	55.72 (2)
Ti2^iv^—Ti1—Ti2^x^	61.730 (7)	Ni1^xxiii^—Ti2—Ti2^xix^	115.62 (3)
Ti2^v^—Ti1—Ti2^x^	118.270 (7)	Ni1^vi^—Ti2—Ti2^xix^	149.263 (5)
Ti1^vi^—Ni1—Ti1^v^	107.95 (2)	Ni1—Ti2—Ti2^xix^	112.73 (2)
Ti1^vi^—Ni1—Ti1^ii^	107.95 (2)	Ti1^ii^—Ti2—Ti2^xix^	149.135 (4)
Ti1^v^—Ni1—Ti1^ii^	107.95 (2)	Ti1^v^—Ti2—Ti2^xix^	100.245 (14)
Ti1^vi^—Ni1—Ti2^xi^	125.12 (2)	Ti2^xviii^—Ti2—Ti2^xix^	60.0
Ti1^v^—Ni1—Ti2^xi^	70.38 (3)	C1^xvi^—Ti2—Ti2^xx^	104.34 (3)
Ti1^ii^—Ni1—Ti2^xi^	125.12 (2)	C1^xvii^—Ti2—Ti2^xx^	45.58 (2)
Ti1^vi^—Ni1—Ti2^xii^	125.12 (2)	Ni1^xxii^—Ti2—Ti2^xx^	115.62 (3)
Ti1^v^—Ni1—Ti2^xii^	125.12 (2)	Ni1^xxiii^—Ti2—Ti2^xx^	55.72 (2)
Ti1^ii^—Ni1—Ti2^xii^	70.38 (3)	Ni1^vi^—Ti2—Ti2^xx^	112.73 (2)
Ti2^xi^—Ni1—Ti2^xii^	68.57 (5)	Ni1—Ti2—Ti2^xx^	149.262 (5)
Ti1^vi^—Ni1—Ti2^xiii^	70.38 (3)	Ti1^ii^—Ti2—Ti2^xx^	100.245 (14)
Ti1^v^—Ni1—Ti2^xiii^	125.12 (2)	Ti1^v^—Ti2—Ti2^xx^	149.135 (4)
Ti1^ii^—Ni1—Ti2^xiii^	125.12 (2)	Ti2^xviii^—Ti2—Ti2^xx^	60.0
Ti2^xi^—Ni1—Ti2^xiii^	68.57 (5)	Ti2^xix^—Ti2—Ti2^xx^	90.001 (1)
Ti2^xii^—Ni1—Ti2^xiii^	68.57 (5)	C1^xvi^—Ti2—Ti2^xxi^	104.34 (3)
Ti1^vi^—Ni1—Ni1^v^	55.837 (19)	C1^xvii^—Ti2—Ti2^xxi^	45.58 (2)
Ti1^v^—Ni1—Ni1^v^	104.31 (2)	Ni1^xxii^—Ti2—Ti2^xxi^	55.72 (2)
Ti1^ii^—Ni1—Ni1^v^	55.837 (19)	Ni1^xxiii^—Ti2—Ti2^xxi^	115.62 (3)
Ti2^xi^—Ni1—Ni1^v^	174.69 (3)	Ni1^vi^—Ti2—Ti2^xxi^	112.73 (2)
Ti2^xii^—Ni1—Ni1^v^	115.62 (3)	Ni1—Ti2—Ti2^xxi^	149.262 (5)
Ti2^xiii^—Ni1—Ni1^v^	115.62 (3)	Ti1^ii^—Ti2—Ti2^xxi^	149.135 (3)
Ti1^vi^—Ni1—Ni1^vi^	104.31 (2)	Ti1^v^—Ti2—Ti2^xxi^	100.245 (14)
Ti1^v^—Ni1—Ni1^vi^	55.837 (19)	Ti2^xviii^—Ti2—Ti2^xxi^	90.0
Ti1^ii^—Ni1—Ni1^vi^	55.837 (19)	Ti2^xix^—Ti2—Ti2^xxi^	60.0
Ti2^xi^—Ni1—Ni1^vi^	115.62 (3)	Ti2^xx^—Ti2—Ti2^xxi^	60.0
Ti2^xii^—Ni1—Ni1^vi^	115.62 (3)	Ti2^xxiv^—C1—Ti2^xxv^	91.17 (4)
Ti2^xiii^—Ni1—Ni1^vi^	174.69 (3)	Ti2^xxiv^—C1—Ti2^xxvi^	88.83 (4)
Ni1^v^—Ni1—Ni1^vi^	60.0	Ti2^xxv^—C1—Ti2^xxvi^	180.0
Ti1^vi^—Ni1—Ni1^ii^	55.837 (19)	Ti2^xxiv^—C1—Ti2^xxvii^	91.17 (4)
Ti1^v^—Ni1—Ni1^ii^	55.837 (19)	Ti2^xxv^—C1—Ti2^xxvii^	88.83 (4)
Ti1^ii^—Ni1—Ni1^ii^	104.31 (2)	Ti2^xxvi^—C1—Ti2^xxvii^	91.17 (4)
Ti2^xi^—Ni1—Ni1^ii^	115.62 (3)	Ti2^xxiv^—C1—Ti2^xxviii^	88.83 (4)
Ti2^xii^—Ni1—Ni1^ii^	174.69 (3)	Ti2^xxv^—C1—Ti2^xxviii^	91.17 (4)
Ti2^xiii^—Ni1—Ni1^ii^	115.62 (3)	Ti2^xxvi^—C1—Ti2^xxviii^	88.83 (4)
Ni1^v^—Ni1—Ni1^ii^	60.0	Ti2^xxvii^—C1—Ti2^xxviii^	180.0
Ni1^vi^—Ni1—Ni1^ii^	60.0	Ti2^xxiv^—C1—Ti2^xxix^	180.0
Ti1^vi^—Ni1—Ti2^xiv^	65.185 (5)	Ti2^xxv^—C1—Ti2^xxix^	88.83 (4)
Ti1^v^—Ni1—Ti2^xiv^	166.08 (5)	Ti2^xxvi^—C1—Ti2^xxix^	91.17 (4)
Ti1^ii^—Ni1—Ti2^xiv^	65.185 (5)	Ti2^xxvii^—C1—Ti2^xxix^	88.83 (4)
Ti2^xi^—Ni1—Ti2^xiv^	123.55 (5)	Ti2^xxviii^—C1—Ti2^xxix^	91.17 (4)
Ti2^xii^—Ni1—Ti2^xiv^	65.437 (12)		

Symmetry codes: (i) *x*−1/4, −*y*, *z*−1/4; (ii) −*x*+1/4, *y*, −*z*+1/4; (iii) −*x*, *y*−1/4, *z*−1/4; (iv) *x*−1/4, *y*−1/4, −*z*; (v) −*x*+1/4, −*y*+1/4, *z*; (vi) *x*, −*y*+1/4, −*z*+1/4; (vii) −*z*, *x*−1/4, *y*−1/4; (viii) *z*, −*x*+1/4, −*y*+1/4; (ix) *y*−1/4, −*z*, *x*−1/4; (x) −*y*+1/4, *z*, −*x*+1/4; (xi) *y*+1/4, −*z*+1/2, *x*−1/4; (xii) −*z*+1/2, *x*−1/4, *y*+1/4; (xiii) *x*−1/4, *y*+1/4, −*z*+1/2; (xiv) *y*, *z*, *x*; (xv) *z*, *x*, *y*; (xvi) *x*, −*y*+3/4, −*z*+3/4; (xvii) *x*, *y*−1/2, *z*−1/2; (xviii) −*y*+3/4, *z*, −*x*+3/4; (xix) *z*+1/2, −*x*+3/4, −*y*+1/4; (xx) *z*+1/2, *x*−1/2, *y*; (xxi) *y*+1/2, *z*, *x*−1/2; (xxii) *x*+1/4, −*y*+1/2, *z*−1/4; (xxiii) *x*+1/4, *y*−1/4, −*z*+1/2; (xxiv) −*y*+1/2, −*z*+1/2, −*x*+1; (xxv) *x*, *y*+1/2, *z*+1/2; (xxvi) −*x*+1, −*y*+1/2, −*z*+1/2; (xxvii) *z*+1/2, *x*, *y*+1/2; (xxviii) −*z*+1/2, −*x*+1, −*y*+1/2; (xxix) *y*+1/2, *z*+1/2, *x*.
