# Supplementary material for: Crystal structure of Ti4Ni2C
Source: IUCrdata. 2024 Jan 19;9(Pt 1):x240043. doi: 10.1107/S2414314624000439 (PMC10842284; doi:10.1107/S2414314624000439)
Supplement: Supplementary file 3 [file x-09-x240043-sup3.docx]

**SUPPLEMENTARY MATERIALS:**

**Crystal structure of Ti_4_Ni_2_C**

**Huizi Liu**^a^, **Xinyu Liang**^a^, **Yibo Liu**^a^**, Changzeng Fan**^a,b,^***, Bin Wen**^a^ **and Lifeng Zhang**^c^

^a^ State Key Laboratory of Metastable Materials Science and Technology, Yanshan University,

Qinhuangdao 066004, People’s Republic of China

^b^ Hebei Key Lab for Optimizing Metal Product Technology and Performance, Yanshan University, Qinhuangdao, Hebei 066004, People’s Republic of China

^c^ School of Mechanical and Materials Engineering, North China University of Technology, Beijing 100144, People’s Republic of China

*Correspondence email: [chzfan@ysu.edu.cn](mailto:chzfan@ysu.edu.cn)

The chemical compositions were examined quantitatively by Oxford spectroscopy (EDX) analysis attached to a ZEISS Sigma 300 field emission SEM for the purpose of guiding the crystal structure refinement. The examined points and areas are designated in Fig. S1, and the corresponding results are listed in Table S1. The deviation relative to the results of refinement of chemical composition is probably caused by the tilt of the single crystal surface to the incident beam. In addition, the conductive adhesives and glues may also result in the detected impurity elements of carbon. For ease of reading, the atomic ratio of Ti, Ni and C was calculated and shown in the last column of Table S1.


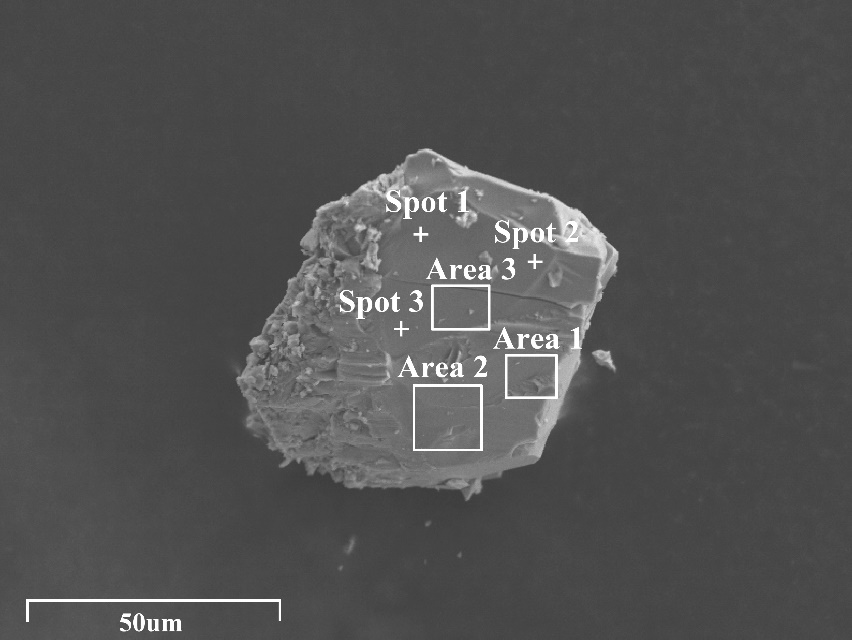


Figure S1 Single crystal of Ti_4_Ni_2_C with selected spots and areas for EDX analysis

**Table S1 EDX results for selected points and areas as designated in Figure S1**

|  | Element | Atomic (%) | Ti : Ni : C |
| --- | --- | --- | --- |
| Spot1 | Ti | 67.1 | 4.08:2:× |
|  | Ni | 32.9 |  |
| Spot2 | Ti | 66.9 | 4.04:2:× |
|  | Ni | 33.1 |  |
| Spot3 | Ti | 67.0 | 4.06:2:× |
|  | Ni | 33.0 |  |
| Area1 | Ti | 47.8 | 4.06:2:2.42 |
|  | C | 28.6 |  |
|  | Ni | 23.6 |  |
| Area2 | Ti | 45.7 | 4.04:2:2.81 |
|  | C | 31.7 |  |
|  | Ni | 22.6 |  |
| Area3 | Ti | 51.1 | 3.94:2:1.78 |
|  | Ni | 25.9 |  |
|  | C | 23.0 |  |
